# Supplementary material for: Impact of the COVID-19 Outbreak—Delayed Referral of Colorectal and Lung Cancer in Primary Care: A National Retrospective Cohort Study
Source: Cancers (Basel). 2023 Feb 25;15(5):1462. doi: 10.3390/cancers15051462 (PMC10000463; doi:10.3390/cancers15051462)
Supplement: Supplementary file 1 [file cancers-15-01462-s001.zip › cancers-2218372-supplementary.pdf]

Impact of the COVID-19 Outbreak—Delayed Referral of Colorectal and Lung Cancer in Primary Care: a National Retrospective Cohort Study

Supplementary materials

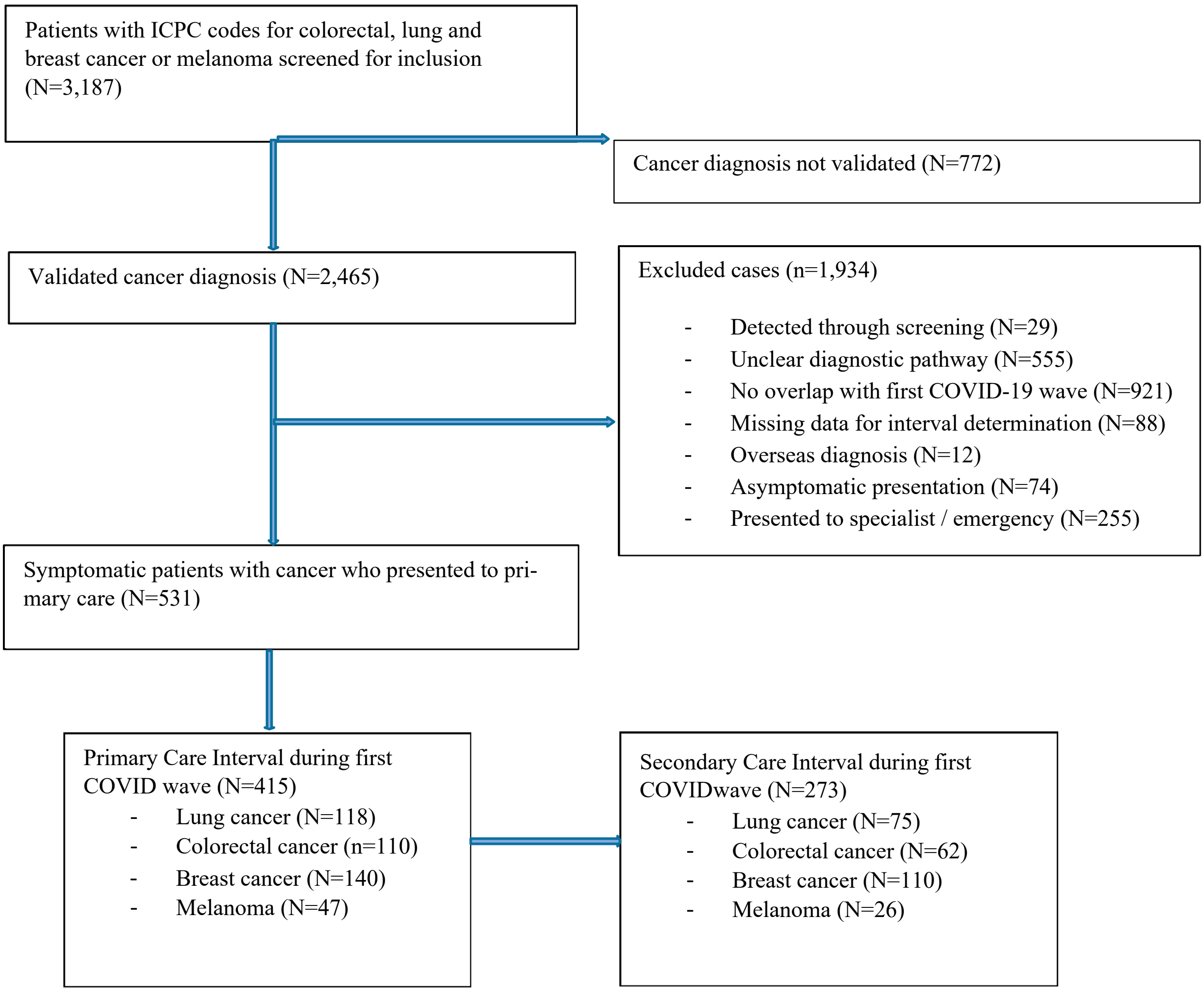

Supplementary flow diagram (Scheme S1)– Patient inclusion for patients diagnosed with cancer during first COVID-19 wave

Supplementary Table S1. Duration of the Secondary Care Interval (ISC); time between GP referral and histological diagnosis, before- and during the first COVID-19 wave for colorectal-, lung-, breast cancer and melanoma.

**Colorectal cancer**					
	**Pre-COVID-19**		**COVID-19**		
	**N**	**Median (IQR)**	**N**	**Median (IQR)**	**P for change**
**Total**	**259**	**19 (8-35)**	**62**	**17.5 (9-52)**	**0.377**
**Gender**					
Male	129	19 (9.5-33.5)	29	16 (9-54)	0.570
Female	130	19 (7-36)	33	18 (9.5-50)	0.515
**Age**					
<65	85	16 (8-44.5)	22	19.5 (11-52)	0.365
>=65	174	19 (8-33)	39	17 (8-67)	0.802
**Comorbidity ***					
<2	50	18.5 (8-38)	19	25 (13-58)	0.173
More than 2	207	19 (8-34)	43	16 (8-35)	0.998
**Psychiatric comorbidity**					
Yes	66	15 (6-34)	13	18 (6-68)	0.801
no	190	19 (9-35)	49	17 (10-48.5)	0.535
**Alarm symptom at first consult**					
Yes	147	20 (10-38)	21	25 (8-52.5)	0.829
No	112	15 (7-30)	41	17 (10-46)	0.152
**Number consults in year prior**					
5 or more	103	19 (8-34)	32	30 (10-75)	0.055
Less than 5	86	14 (8-36.5)	30	15.5 (9-25)	0.815
**Lung cancer**					
	**Pre-COVID-19**		**COVID-19**		
	**N**	**Median (IQR)**	**N**	**Median (IQR)**	**P for change**
**Total**	**197**	**18 (8-35)**	**75**	**18 (7-40)**	**0.978**
**Gender**					
Male	110	18.5 (8-34)	33	16 (7-45)	0.854
Female	87	17 (9-36)	42	18.5 (7-36)	0.964
**Age**					
<65	69	18 (9-32.5)	30	19 (7-40)	0.656
>=65	128	17 (8-35)	45	16 (6-40)	0.687
**Comorbidity ***					
Less than 2	45	15 (7-33)	18	13.5 (7-20)	0.488
2 or more	149	18 (8-35)	47	23 (7-42)	0.445
**Psychiatric comorbidity**					
Yes	40	16 (8.25-30.5)	14	40 (14-48.5)	0.042
no	154	18 (8-35)	61	16 (7-31)	0.415
**Alarm symptom at first consult**					
Yes	43	16 (8-31)	13	18 (6.5-43.5)	0.560
No	154	18.5 (8-36)	62	17.5 (7-40)	0.837
**Number consults in year prior**					
5 or more	100	17.5 (8-36)	50	24 (11-45)	0.351
Less than 5	57	17 (7-36)	25	15 (6-23)	0.238
**Breast cancer**					
	**Pre-COVID-19**		**COVID-19**		
	**N**	**Median (IQR)**	**N**	**Median (IQR)**	**P for change**
**Total**	**256**	**3 (2-7)**	**110**	**6 (3-9)**	**<0.001**
**Age**					

<65	162	3 (2-6)	68	6 (3-9)	<0.001
>=65	94	4 (2-7)	42	5 (2-8)	0.309
**Comorbidity ***					
<2	125	3 (2-7)	59	5 (3-9)	0.040
More than 2	131	3 (2-7)	51	6 (4-9)	0.000
**Psychiatric comorbidity**					
Yes	44	3 (2-7)	32	6 (3-9)	0.012
no	210	3 (2-7)	78	5.5 (3-9)	<0.001
**Alarm symptom at first consult**					
Yes	243	3 (2-7)	97	5 (3-8.5)	<0.001
No	13	6 (2.5-20)	13	8 (6-32.5)	0.257
**Number consults in year prior**					
5 or more	88	3 (2-7)	64	5.5 (3-8)	0.013
Less than 5	117	3 (2-7)	46	6 (3-10.5)	<0.001
			**Melanoma**		
	**Pre-COVID-19**		**COVID-19**		
	**N**	**Median (IQR)**	**N**	**Median (IQR)**	**P for change**
**Total**	**105**	**14 (7-29)**	**26**	**9 (3-43.5)**	**0.441**
**Gender**					
Male	54	12.5 (5-23.5)	12	9 (2-31)	0.549
Female	51	15.5 (7-34)	14	9 (3.5-78.5)	0.604
**Age**					
<65	69	14 (5.5-33.5)	13	11 (3-47)	0.869
>=65	37	12 (7.5-21.5)	13	7 (2.5-66.5)	0.319
**Comorbidity ***					
<2	50	14 (4.75-33)	15	6 (2-22)	0.210
More than 2	56	12.5 (8-27)	11	20 (4-99)	0.576
**Psychiatric comorbidity**					
Yes	19	16 (7-35)	2	60.5 *(IQR n.a.)*	n.a.
no	87	13 (6-28)	24	6.5 (2-39)	0.297
**Alarm symptom at first consult**					
Yes	81	14 (6-30.5)	21	7 (2.5-63)	0.691
No	25	14 (7-28.5)	5	11 (3-26)	0.290
**Number consults in year prior**					
5 or more	38	9 (6-25)	14	4 (2-34.5)	0.211
Less than 5	42	13 (5-27)	12	16 (6-50.5)	0.417

P. for diff.: P-value for difference in durations pre-COVID-19 and during first COVID-19 wave. No significance testing for differences in COVID-19 impact between subgroups within cancer populations was performed, because of low N. IQR: Interquartile Range. Periods: Pre-COVID-19: 2012-2015 - first COVID-19 wave: March-June 2020. *Comorbidity was defined in accordance with O'Halloran J, Miller GC, Britt H. Defining chronic conditions for primary care with ICPC-2. Family practice. 2004;21(4):381-6.
